# Heterogeneous Nucleation in Solutions on Rough Solid Surfaces: Generalized Gibbs Approach

**DOI:** 10.3390/e21080782

**Published:** 2019-08-09

**Authors:** Alexander S. Abyzov, Leonid N. Davydov, Jürn W. P. Schmelzer

**Affiliations:** 1 National Science Center Kharkov Institute of Physics and Technology, 61108 Kharkov, Ukraine; 2 Institute of Physics, University of Rostock, Albert-Einstein-Strasse 23-25, 18059 Rostock, Germany

**Keywords:** heterogeneous nucleation, kinetic theory, rough surface, Gibbs theory, surface tension, 64.60.Bd General theory of phase transitions, 64.60.Q Nucleation in phase transitions, 82.60.Nh Thermodynamics of nucleation, 68.35.Md Surface energy of surfaces and interfaces, 64.60.an Phase transitions in finite-size systems, 68.35.Md Thermodynamic properties of surfaces and interfaces

## Abstract

Heterogeneous nucleation of new phase clusters on a rough solid surface is studied. The ambient phase is considered to be a regular supersaturated solution. In contrast to existing studies of the same problem, the possible difference between the state parameters of the critical cluster and the corresponding parameters of a newly formed macroscopic phase is accounted for. This account is performed within the framework of the generalized Gibbs approach. Surface imperfections are chosen in the form of cones. The model allows us to simplify the analysis but also to obtain the basic results concerning the defect influence on the nucleation process. It is shown that the catalytic activity factor for nucleation of the cone depends both on the cone angle and the supersaturation in the solution determining the state parameters of the critical clusters. Both factors considerably affect the work of critical cluster formation. In addition, they may even lead to a shift of the spinodal curve. In particular, in the case of good wettability (macroscopic contact angle is less than 90°) the presence of surface imperfections results in a significant shifting of the spinodal towards lower values of the supersaturation as compared with heterogeneous nucleation on a planar solid surface. With the decrease of the cone pore angle, the heterogeneous spinodal is located nearer to the binodal, and the metastability range is narrowed, increasing the range of states where the solution is thermodynamically unstable.

## 1. Introduction

The nucleation of new phase clusters can be catalyzed by solid or liquid particles dissolved in the ambient phase, by planar surfaces, and, in particular, by defects of such surfaces. In all these cases of heterogeneous nucleation the thermodynamic barrier—the work of formation of the critical cluster which must be overcome for a nucleus for consequent deterministic growth—is reduced as compared to homogeneous nucleation when the surface or particles dissolved in the ambient phase are absent. Such effects are intensively studied in the framework of classical nucleation theory [1,2,3,4,5,6]. However, the classical theory of nucleation (both homogeneous, and heterogeneous) commonly relies on the assumption that the state parameters of the critical cluster are widely identical to the corresponding parameters of the macroscopic phase to be formed. However, in practice this assumption, as a rule, is not met [7,8,9,10]. The significance of such changes of the state parameters of the critical clusters in heterogeneous nucleation was demonstrated by us for the first time in Refs. [11,12,13]. In [11,12], we studied such processes for condensation and boiling on planar interfaces, in [13], we considered condensation and boiling on rough interfaces. This analysis is extended here considering heterogeneous nucleation in solutions catalyzed by rough solid interfaces.

In detail, in the present paper a theoretical analysis of heterogeneous nucleation in a binary regular solution on a rough solid surface is conducted employing the generalized Gibbs approach. The main difference of the proposed approach from theoretical treatments performed so far consists, as already noted above, is in the consistent account of the difference between the state parameters of the critical cluster and the corresponding parameters of the macroscopic phase to be formed. Surface imperfections are chosen in form of cones that allows us to simplify the analysis and at the same time to obtain the main results of the defect influence on the nucleation process. The general qualitative conclusions do not depend widely on the specific model employed for the description.

The thermodynamic analysis of nucleation in terms of the generalized Gibbs approach supplies us with the work of formation of the aggregates of the newly evolving phase in dependence on size and shape. This dependence we analyze here. The knowledge of such dependence is a precondition for modeling the kinetics of both nucleation and growth processes. In nucleation and growth, the clusters may change both their shape and size and both parameters may even fluctuate. However, this analysis refers to a different topic and will be addressed in a future study.

## 2. Basic Equations

We consider the formation of a new phase cluster on a rough rigid surface. For the description of the bulk properties of the ambient and the newly formed phases we use the model of a binary solid or liquid regular solution. The chemical potentials $\mu_{j}$ of each of the two components (j=1,2) of a regular solution can be written in the form [14]

(1) $$\mu_{1}=\mu_{1}^{*}+k_{B}T\ln\left(1-x\right)+\Omega x^{2}\;,$$

(2) $$\mu_{2}=\mu_{2}^{*}+k_{B}T\ln x+\Omega {\left(1-x\right)}^{2}\;,$$

where $k_{B}$ is the Boltzmann constant, *T* is the absolute temperature, *x* and (1−x) are the molar fractions of the second and first components, correspondingly (for unambiguity we consider the solvent as the first component and the dissolved substance as the second component), $\Omega =2k_{B}T_{c}$ is the interaction parameter describing specific properties of the considered system, and $T_{c}$ is the critical temperature of the system.

In thermodynamics, the binodal curve is the locus of phase states (in (T,x)-diagram) where two distinct phases may coexist in equilibrium. This coexistence curve is defined by the condition at which the chemical potentials of solution components are equal in each phase. The extremum of the binodal curve in temperature is known as a critical point. At this point, the binodal curve coincides with the extremum of the spinodal curve. The spinodal curve in its turn is the locus of the phase states where the system’s local stability with respect to small fluctuations is broken and is defined by the condition that the second derivative of Gibbs free energy (with respect to concentration *x*) is zero. Therefore, in our case at constant external pressure the positions of the binodal and spinodal on the phase diagram (T,x) are determined by the following equations,

(3) $$\ln\left(\frac{1-x}{x}\right)=2\frac{T_{c}}{T}\left(1-2x\right)\;,$$

(4) $$x\left(1-x\right)=\frac{T}{4T_{c}}\;.$$

**Figure 1:**
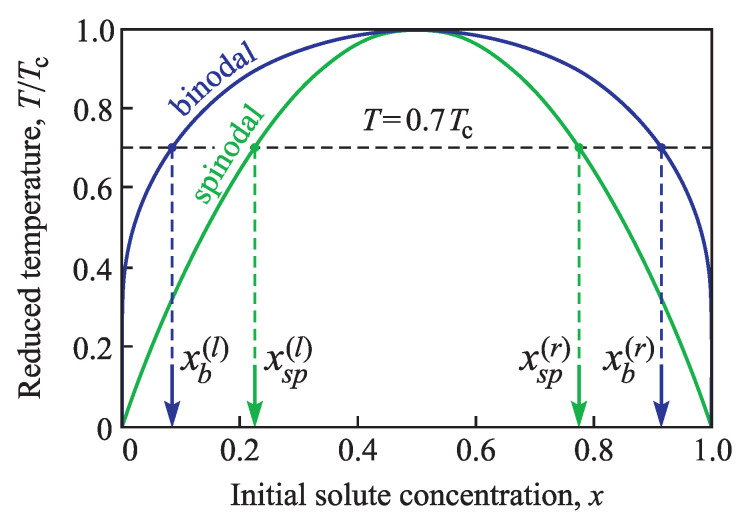
Binodal (dark blue) and spinodal (green) curves as functions of the composition of a regular solution. The left and right binodal ($x_{b}^{\left(l\right)}$, $x_{b}^{\left(r\right)}$) and spinodal ($x_{sp}^{\left(l\right)}$, $x_{sp}^{\left(r\right)}$) values are shown for temperature $T=0.7T_{c}$.

They are shown in Figure 1.

The values of the left binodal ($x_{b}^{\left(l\right)}$) and spinodal ($x_{sp}^{\left(l\right)}$) branches, calculated at temperature $T=0.7T_{c}$, are, correspondingly

(5) $$x_{b}^{\left(l\right)}=0.1857\;,\;x_{sp}^{\left(l\right)}=0.2261\;.$$

Both curves are symmetric with respect to x=1/2; therefore, the corresponding values for the right-hand side branches are

(6) $$x_{b}^{\left(r\right)}=1-x_{b}^{\left(l\right)}=0.9143\;,\;x_{sp}^{\left(r\right)}=1-x_{sp}^{\left(l\right)}=0.7739\;.$$

These values are especially distinguished in Figure 1.

Let us assume that due to the change in temperature or composition, the system is transferred into a metastable state somewhere between left binodal and spinodal ($x_{b}^{\left(l\right)}<x<x_{sp}^{\left(l\right)}$, see Figure 1). After this sudden transfer, temperature and composition are maintained unchanged. For this system first we define the parameters of the critical cluster nucleated in a conic pore depending on the created supersaturation (i.e., the molar concentration of the dissolved substance, *x*). We remind that as an example of surface imperfections we choose a conic pore. This approach allows us to simplify the analysis and at the same time to receive main results of the defect influence on the nucleation process.

**Figure 2:**
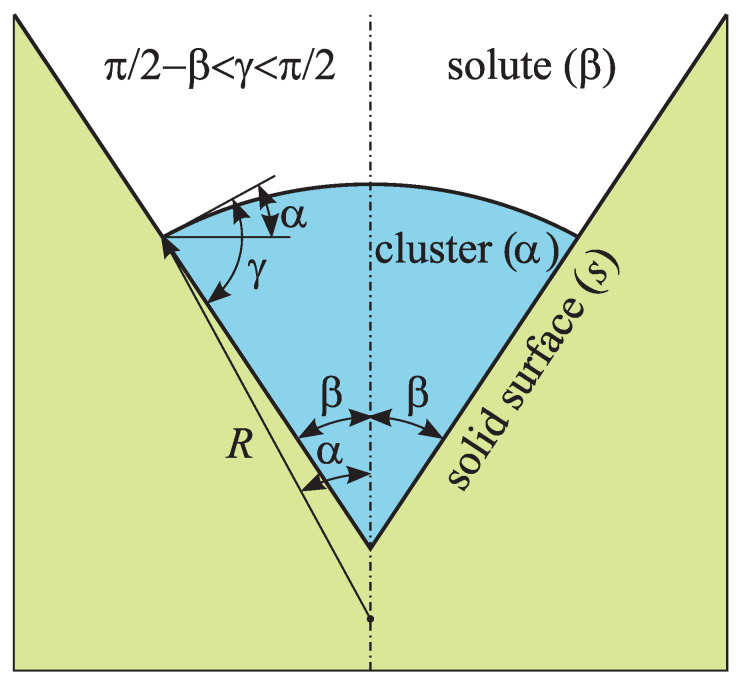
Model used in the analysis of heterogeneous nucleation of a new phase cluster in a conic pore. Here *R* is the curvature radius of the cluster outer surface, γ is the contact angle, and 2β is the cone angle.

In a binary solution a new phase will be nucleated as a result of the redistribution of molecules (atoms) in space. Following Gibbs model [6], we consider a new phase cluster as a spatially homogeneous part of the system with a composition, however, different from the ambient phase. The boundary is modeled by a mathematical surface of zero thickness with a corresponding value of the tension surface [1,6,12]. The change of the thermodynamic potential (Gibbs free energy *G*) of the binary system owing to the creation of a cluster in form of a spherical cone with the radius *R* in a conic pore (Figure 2) can be given by [1,2,3,11]

(7) $$\Delta G=\sigma_{\alpha \beta }A_{\alpha \beta }+\left(\sigma_{\alpha s}-\sigma_{\beta s}\right)A_{\alpha s}+\sum_{j=1,2}n_{j}\left(\mu_{j\alpha }-\mu_{j\beta }\right)\;.$$

Here specific interphase energies (surface tensions) of the corresponding boundaries are denoted as: $\sigma_{\alpha s}$ (cluster (α)–pore (*s*)), $\sigma_{\beta s}$ (outer solution (β)–pore (*s*)), and $\sigma_{\alpha s}$ (cluster (α)–outer solution (β)). Next, $A_{\alpha s}$ and $A_{\alpha \beta }$ are the boundary surface area between the cluster and pore, and the outer solution, correspondingly (Figure 2), $\mu_{j\alpha }$ and $\mu_{j\beta }$ are the chemical potentials of both components (j=1,2) in the cluster and outside it (see Equations (1) and (2)). The indices α and β denote the parameters of the cluster and the ambient phase, accordingly. For the description of the cluster state the numbers of atoms of a kind 1 and 2 are used as independent variables, $n_{1}$ and $n_{2}$ (the index α in $n_{1}$ and $n_{2}$ is omitted to simplify the notations). The total number of atoms in a cluster is $n_{\alpha }=n_{1}+n_{2}$.

For simplification, similarly to [10,12], the particle volume ω is supposed not to depend on composition ($\omega_{\alpha }=\omega_{\beta }\equiv \omega =a^{3}$, where *a* is the interatomic distance).

The radius of curvature, *R*, of the spherical cone (for simplicity we will name it “cluster radius”) is determined by the number of particles in the cluster, $n_{\alpha }$, via

(8) $$\varphi \frac{4\pi }{3}R^{3}=n_{\alpha }\omega =n_{\alpha }a^{3}\;,$$

where φ is expressed through the contact angle, γ, and the cone angle, 2β, as

(9) $$\varphi =\frac{1}{3}\left(2-3\cos\alpha +\cos^{3}\alpha +\cot\beta \sin^{3}\alpha \right)\;,\;\alpha =\gamma +\beta -\frac{\pi }{2}\;.$$

The change in Gibbs free energy due to cluster creation is determined in correspondence with Equation (7) as [2,3,11]

(10) $$\Delta G=\Delta G_{V}+\Delta G_{S}\;,$$

(11) $$\Delta G_{V}=-\varphi \left(\frac{4\pi }{3\omega }\right)R^{3}\Delta \mu =\varphi \left(\frac{4\pi }{3\omega }\right)R^{3}k_{B}Tf\;,\;\Delta \mu =-k_{B}Tf\;,$$

(12) $$\Delta G_{S}=2\pi R^{2}\left(1-\cos\alpha \right)\sigma_{\alpha \beta }+\pi R^{2}\frac{1-\cos^{2}\alpha }{\sin\beta }\left(\sigma_{\alpha s}-\sigma_{\beta s}\right)\;.$$

For a wettable surface (γ<π/2) at low cone angle of β<π/2−γ the cluster outer surface becomes concave, the contribution of the surface component in the work of its formation becomes negative, and the cluster can start to grow in the range $x>x_{b}$ at any initial size. However, this conclusion is correct only for a conic pore which has a sufficiently large depth. Indeed, when the cluster grows up to a flat surface surrounding the pore its surface becomes convex, and, actually, one must consider nucleation on a flat surface [12]. If a pore is not deep, the cluster does not succeed to grow up to a critical size, and the effect of the pore decreases. This particular case is beyond the scope of the present work, therefore we shall limit ourselves here to the range of angles β>π/2−γ (see Figure 2).

In the derivation of Equation (11) we have neglected possible modifications of the solution composition caused by the nucleation process. This effect is not essential at an early stage of nucleation for sufficiently large systems. An analysis of the effect of such changes in systems of small sizes is given in [10,15]. At such conditions, the function $f(x_{\alpha },x)$ in Equation (11) has the meaning of the thermodynamic driving force of cluster formation. It is determined by the relation [2,3,11],

(13) $$f\left(x_{\alpha },x\right)=\left(1-x_{\alpha }\right)\left\{\ln\frac{1-x_{\alpha }}{1-x}+2\frac{T_{c}}{T}\left(x_{\alpha }^{2}-x^{2}\right)\right\}+x_{\alpha }\left\{\ln\frac{x_{\alpha }}{x}+2\frac{T_{c}}{T}\left[{\left(1-x_{\alpha }\right)}^{2}-{\left(1-x\right)}^{2}\right]\right\}\;.$$

**Figure 3:**
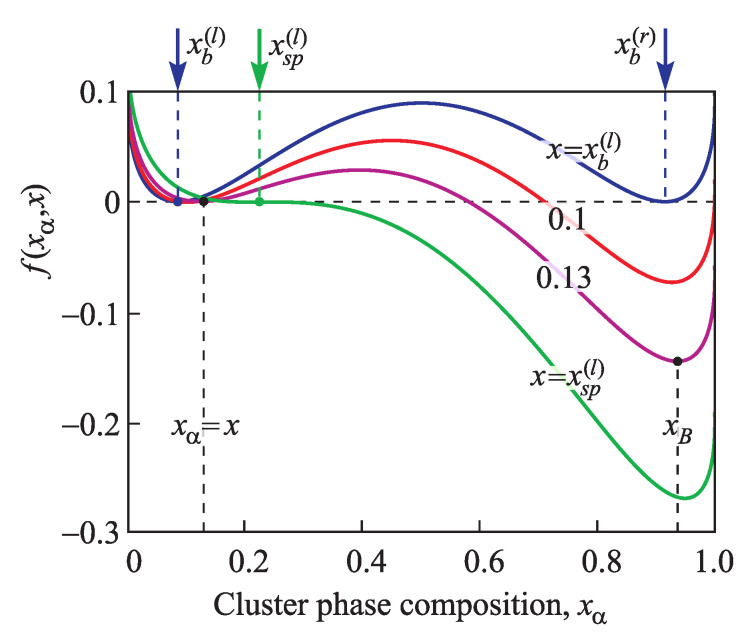
Dependence of the function $f(x_{\alpha },x)$ on cluster composition, $x_{\alpha }$, at different supersaturations $x=x_{b}^{\left(l\right)},\;0.1,\;0.13,\;x_{sp}^{\left(l\right)}$ in the region of metastability $x_{b}^{\left(l\right)}<x<x_{sp}^{\left(l\right)}$ of the ambient solution.

The dependence of function $f(x_{\alpha },x)$ on cluster composition, $x_{\alpha }$, for different values of the supersaturations, *x*, is shown in Figure 3.

The regions of metastability are in composition ranges $x_{b}^{\left(l\right)}<x<x_{sp}^{\left(l\right)}$ (between the left branches of the binodal and spinodal) and $x_{sp}^{\left(r\right)}<x<x_{b}^{\left(r\right)}$ (between the right branches of the spinodal and binodal). The function $f(x_{\alpha })$ has one maximum and two minima (for $x\neq x_{sp}^{\left(l,r\right)}$). The first minimum, $x_{\alpha }=x$, corresponds to the state of the ambient phase. The second minimum, $x_{\alpha }=x_{B}$, corresponds to the final macroscopic state of the precipitating phase, to which a cluster evolves at fixed composition of the surrounding solution, *x*. It is determined by the minimum of the bulk contribution to the Gibbs free energy (Figure 3),

(14) $${\left.\frac{\partial f(x_{\alpha },x)}{\partial x_{\alpha }}\right|}_{x=x_{B}}=0.$$

At the spinodal, $x=x_{sp}^{\left(l\right)}$, the function $f(x_{\alpha })$ has an inflection point corresponding to $x_{\alpha }=x$, ${\left.\frac{\partial^{2}f\left(x_{\alpha },x\right)}{\partial x_{\alpha }^{2}}\right|}_{x_{\alpha }=x}=0$. The range $x_{sp}^{\left(r\right)}<x<x_{sp}^{\left(l\right)}$ is thermodynamically unstable. The maximum of the function $f(x_{\alpha })$ in this region corresponds to the initial state, $x_{\alpha }=x$, there are also two local minima of the function $f(x_{\alpha })$ at $x_{\alpha }=x_{A}<x$ and $x_{\alpha }=x_{B}>x$. Similar to Equation (14) they are determined by the equation

(15) $${\left.\frac{\partial f(x_{\alpha },x)}{\partial x_{\alpha }}\right|}_{x=x_{A}\;\mathrm{or}\;x=x_{B}}=0\;.$$

**Figure 4:**
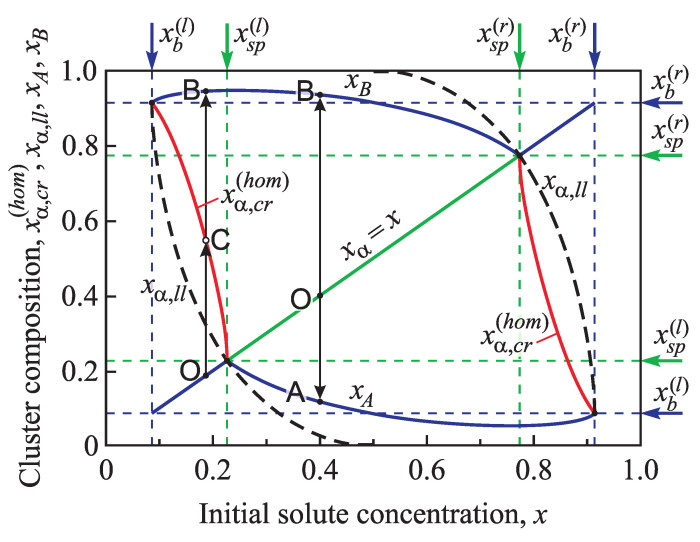
Composition $x_{\alpha ,cr}^{\left(\mathrm{hom}\right)}$ of the critical cluster (red lines), its minimum value $x_{\alpha ,ll}$ (dotted line), and concentrations $x_{A},\;x_{B}$ (continuous dark blue lines) in dependence on the initial composition, *x*, of the solution. These results refer to the case of homogeneous nucleation.

Figure 4 illustrates the dependence of the concentrations $x_{A}$ and $x_{B}$ on the initial composition *x* of the surrounding solution in the whole possible range of compositions. Taking into account the symmetry with respect to the substitution x↔1−x, we consider only initial states with a composition x≤1/2.

At any given pressure and temperature, the thermodynamic driving force for cluster formation should be positive, i.e., $f(x_{\alpha },x)<0$ (the bulk contribution to Gibbs free energy is decreased in this case [11]). This condition holds for

(16) $$x_{\alpha }<x_{\alpha ,ll},$$

where $x_{\alpha ,ll}$ is the solution of the equation

(17) $$f\left(x_{\alpha ,ll},x\right)=0.$$

The function $x_{\alpha ,ll}\left(x\right)$ is represented in Figure 4 by a dashed line, and the composition of the critical cluster for homogeneous nucleation $x_{\alpha ,cr}^{\left(\mathrm{hom}\right)}$ is shown by a solid line. The evolution of an initially metastable state proceeds along the following path: O→C→B. It starts at the initial state $x_{\alpha }=x$ and propagates through a critical cluster (**C**) to $x_{B}$. For an initially unstable state two variants of evolution are possible, first O→A, with a decrease of the cluster concentration to $x_{A}$, and second O→B, with an increase in the concentration up to $x_{B}$. Generally, the inequality $x_{\alpha ,ll}\leq x_{\alpha ,cr}^{\left(\mathrm{hom}\right)}$ holds, it goes over to an equality only at $x=x_{b}^{\left(l\right)}$ and $x=x_{sp}^{\left(l\right)}$. In the latter case, when $x_{\alpha ,ll}=x_{\alpha ,cr}^{\left(\mathrm{hom}\right)}$, the cluster can evolve without overcoming a potential barrier. This process corresponds to spinodal decomposition. As will be shown below, at heterogeneous nucleation in a conic pore a similar situation can arise even at appreciably smaller supersaturations $x<x_{sp}^{\left(l\right)}$.

The Young equation determines the mechanical equilibrium at the contact line of three phases [1,2,3,4,5]

(18) $$\sigma_{\beta s}=\sigma_{\alpha s}+\sigma_{\alpha \beta }\cos\gamma \;.$$

Assuming that this condition is fulfilled and taking into account Equation (9) we obtain from Equation (12) the interfacial contribution of a new phase cluster to the Gibbs free energy as

(19) $$\Delta G_{S}=4\pi R^{2}\sigma_{\alpha \beta }\left[\frac{1-\cos\alpha }{2}-\frac{\cos\gamma (1-\cos^{2}\alpha )}{4\sin\beta }\right]=4\pi R^{2}\sigma_{\alpha \beta }\varphi \;.$$

Thus, the work of cluster formation at heterogeneous nucleation in a conic pore can be written as

(20) $$\Delta G_{\mathrm{het}}=\varphi \Delta G_{\mathrm{hom}}\;,$$

where

(21) $$\frac{\Delta G_{\mathrm{hom}}}{k_{B}T}=\frac{4\pi }{3\omega }\left[\frac{3}{2}R_{\sigma }R^{2}{\left(x_{\alpha }-x\right)}^{2}+R^{3}f\left(x_{\alpha },x\right)\right]\;,$$

(22) $$R_{\sigma }=\frac{2\sigma_{\alpha \beta ,0}a^{3}}{k_{B}T}{\left(x_{b}^{\left(r\right)}-x_{b}^{\left(l\right)}\right)}^{-2}\;,$$

and the catalytic factor φ is determined by Equation (9). Equations (19)–(21) are fulfilled at all possible values of the cone pore angle β, contact angle γ, and radius *R* (note, however, that here we do not consider the case of a concave outer surface of the cluster, when R<0). Equation (19) is similar to that obtained for heterogeneous nucleation on a smooth planar surface [11] and differs only by the factor sinβ (which equals unity for a flat surface, when $\beta =90{}^{{}^{\circ}}$). The catalytic factor φ within the framework of the generalized Gibbs approach becomes dependent not only on the angles γ and β, but also on the compositions of the initial phase and the critical cluster. The specific interfacial energy between two phases with compositions $x_{\alpha }$ and *x*, can be expressed according to Becker [14] (see also [16]) as

(23) $$\sigma_{\alpha \beta }=\sigma_{\alpha \beta ,0}{\left(\frac{x_{\alpha }-x}{x_{b}^{\left(r\right)}-x_{b}^{\left(l\right)}}\right)}^{2}.$$

Here $\sigma_{\alpha \beta ,0}$ is the respective value, when a new phase cluster and the solution surrounding it are in equilibrium, i.e., $x=x_{b}^{\left(l\right)}$ and $x=x_{b}^{\left(r\right)}$.

For further analysis it is convenient to introduce the dimensionless variables

(24) $$r\equiv \frac{R}{R_{\sigma }},\;\Delta g\equiv \frac{\Delta G}{G_{\sigma }}\;,$$

(25) $$G_{\sigma }=\frac{16\pi }{3}\frac{{\left(\sigma_{\alpha \beta ,0}a^{2}\right)}^{3}}{{\left(k_{B}T\right)}^{2}}{\left(x_{b}^{\left(r\right)}-x_{b}^{\left(l\right)}\right)}^{-6}\;.$$

In these variables Equation (20) takes the form

(26) $$\Delta g\left(r,x_{\alpha }\right)=\varphi \left(\gamma ,\beta \right)\left[3r^{2}{\left(x_{\alpha }-x\right)}^{2}+2r^{3}f\left(x_{\alpha },x\right)\right].$$

As already was noted above, *R* is the radius of the cluster surface contacting with the ambient solution, and it can have positive, infinite, and negative values. Therefore, it is more convenient to use as independent variables for the description of the cluster state the numbers of atoms in the cluster $\left(n_{1},n_{2}\right)$ instead of (r,x). Also is convenient to normalize these quantities to $n_{\sigma }$ as

(27) $$n_{1}^{\prime }\equiv \frac{n_{1}}{n_{\sigma }}\;,\;n_{2}^{\prime }\equiv \frac{n_{2}}{n_{\sigma }}\;,\;n_{\sigma }\equiv \frac{4\pi }{3}{\left(\frac{R_{\sigma }}{a}\right)}^{3}\;.$$

To simplify the notations we omit primes, then Equation (26) takes the form

(28) $$\Delta g\left(n_{1},n_{2},x\right)=3{\left[\varphi \left(\gamma ,\beta \right)\right]}^{\frac{1}{3}}n^{\frac{2}{3}}{\left(\frac{n_{2}}{n}-x\right)}^{2}+2nf\left(x,\frac{n_{2}}{n}\right)\;,$$

where $n\equiv n_{1}+n_{2}=\varphi r^{3}$. The parameters of the critical cluster, $\left(n_{1,cr},n_{2,cr}\right)$, are determined by a solution of the set of equations

(29) $$\frac{\partial \Delta g(n_{1},n_{2},x)}{\partial n_{1}}=0\;,\;\frac{\partial \Delta g(n_{1},n_{2},x)}{\partial n_{2}}=0\;.$$

The work of formation of the critical cluster is determined by

(30) $$\left(\frac{\Delta G_{cr}}{G_{\sigma }}\right)\equiv \Delta g_{cr}\left(x\right)=\Delta g\left(n_{1,cr},n_{2,cr},x\right)\;.$$

Above relations are the basic for the subsequent analysis of surface roughness on the properties of critical clusters in heterogeneous nucleation on rough surfaces. This analysis we will start with the discussion of the contact angle.

## 3. Determination of the Contact Angle

In classical nucleation theory the parameters of a cluster are taken to be widely equal to the properties of the newly evolving macroscopic phase. By this reason, the values of the specific surface energies in Young’s equation, Equation (18), are constants for some given values of pressure and temperature. Consequently, the contact angle is also constant. In the generalized Gibbs approach, parameters of a new phase cluster are functions of the supersaturation, therefore the contact angle γ and, consequently, the catalytic factor φ(γ,β) also depend on supersaturation.

**Figure 5:**
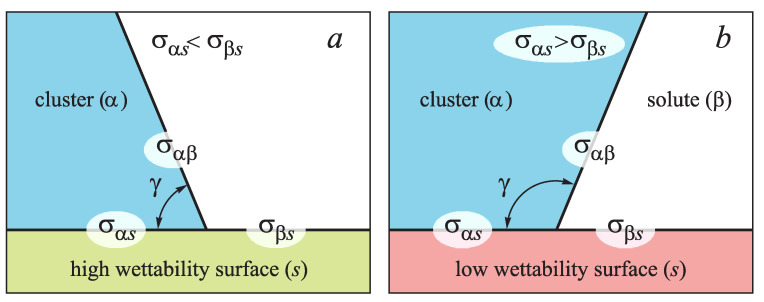
Contact angle γ for well (**a**) and badly (**b**) wettable surfaces. The corresponding specific interface energies are $\sigma_{\alpha \beta },\;\sigma_{\beta s}$, and $\sigma_{\alpha s}$.

For the case when the surface tension of the cluster boundary with the pore surfaces is less than that between solutions and the same surfaces ($\sigma_{\alpha s}<\sigma_{\beta s}$), and the contact angle determined by Young Equation (18), as

(31) $$\cos\gamma =\frac{\sigma_{\beta s}-\sigma_{\alpha s}}{\sigma_{\alpha \beta }},$$

is less than 90${}^{{}^{\circ}}$, the surface is well wettable (Figure 5a). Otherwise, $\sigma_{\alpha s}<\sigma_{\beta s}$, the contact angle is larger than 90${}^{{}^{\circ}}$, the surface is badly wettable (Figure 5b). In the present work we consider only the first case, when wettability is good and the influence of surface defects becomes most apparent.

For the determination of the contact angle one must know the specific interfacial energies of all boundaries as functions of the cluster and surrounding solution compositions (for unification of the notations we shall use the term “fluid” both for cluster and solution and denote it with a subindex “f”) in the whole range from the left binodal, $x_{b}^{\left(l\right)}$, up to the right binodal, $x_{b}^{\left(r\right)}$. It is easy to show that in a simple linear approximation the specific interfacial energy of the fluid-surface interphase can be expressed as (details see [13])

(32) $$\sigma_{fs}\left(\rho \right)=\frac{\sigma_{\beta s,0}\left(x_{b}^{\left(r\right)}-x\right)+\sigma_{\alpha s,0}\left(x-x_{b}^{\left(l\right)}\right)}{x_{b}^{\left(r\right)}-x_{b}^{\left(l\right)}}.$$

Here, as above (see Equation (23)), the index “0” relates to the case, when a new phase cluster and surrounding solution are in equilibrium, i.e., $x=x_{b}^{\left(l\right)}$ and $x_{\alpha }=x_{b}^{\left(r\right)}$, and the quantities without index “0” denote parameters for current composition (cluster or solution). From Equation (32) it follows that

(33) $$\sigma_{\beta s}-\sigma_{\alpha s}=\sigma_{fs}\left(x\right)-\sigma_{fs}\left(x_{\alpha }\right)=\left(\sigma_{\beta s,0}-\sigma_{\alpha s,0}\right)\left(\frac{x_{\alpha }-x}{x_{b}^{\left(r\right)}-x_{b}^{\left(l\right)}}\right)\;.$$

It is evident that the difference $\left(\sigma_{\beta s}-\sigma_{\alpha s}\right)$ is a linear function of $\left(x_{\alpha }-x\right)$. It is positive when $\sigma_{\beta s,0}>\sigma_{\alpha s,0}$ (good wetting) and is negative when $\sigma_{\beta s,0}<\sigma_{\alpha s,0}$ (bad wetting) in correspondence with above-stated definition. This difference determines the degree of catalytic activity of the solid surface at heterogeneous nucleation.

From Equation (23) with allowance for Equations (31) and (33) we obtain an expression determining the contact angle γ as a function of the compositions of the cluster, $x_{\alpha }$, and of the surrounding solution, *x*,

(34) $$\cos\gamma \left(x,x_{\alpha }\right)=\cos\gamma_{0}\left(\frac{x_{b}^{\left(r\right)}-x_{b}^{\left(l\right)}}{x_{\alpha }-x}\right)\;,$$

where

(35) $$\cos\gamma_{0}=\frac{\left(\sigma_{\beta s,0}-\sigma_{\alpha s,0}\right)}{\sigma_{\alpha \beta ,0}}\;.$$

Thus, for the further analysis there is no need in the knowledge of the specific interfacial energies; it merely required to know the equilibrium contact angle $\gamma_{0}$.

In the considered case of cluster nucleation on a well-wettable surface the angle α, defining the catalytic activity factor Equation (9), has values larger zero only when $x_{\alpha }<x_{\alpha ,0}$ where $x_{\alpha ,0}$ is determined by the equation

(36) $$x_{\alpha ,0}=\left(x_{b}^{\left(r\right)}-x_{b}^{\left(l\right)}\right)\frac{\cos\gamma_{0}}{\cos\left(\pi /2-\beta \right)}+x\;.$$

**Figure 6:**
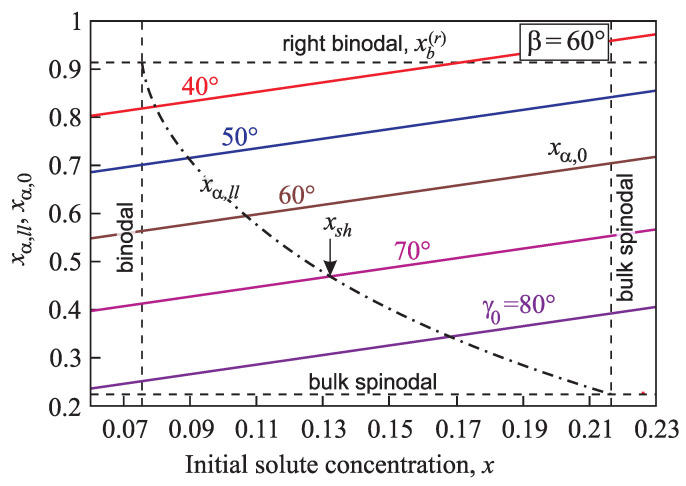
Dependence of function $f(x_{\alpha },x)$ on cluster composition $x_{\alpha }$ at different supersaturations $x=x_{b}^{\left(l\right)},\;0.1,\;0.13,\;x_{sp}^{\left(l\right)}$ in the region of metastability $x_{b}^{\left(l\right)}<x<x_{sp}^{\left(l\right)}$.

Intersection of the plots $x_{\alpha ,0}\left(x\right)$ and $x_{\alpha ,ll}\left(x\right)$ (see Equations (16) and (17) and Figure 6) determines the position of the spinodal $x_{sh}$ for heterogeneous nucleation

(37) $$x_{\alpha ,0}\left(x_{sh}\right)=x_{\alpha ,ll}\left(x_{sh}\right)\;.$$

**Figure 7:**
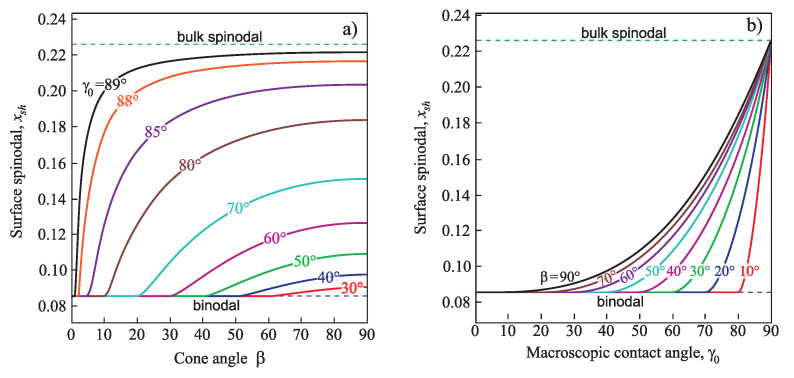
Dependence of the spinodal position $x_{sh}$ for heterogeneous nucleation on cone angle, β, (**left**) and equilibrium contact angle, $\gamma_{0}$ (**right**).

At $x>x_{sh}$ the catalytic activity factor equals zero, φ(γ,β)=0, i.e., in this case the nucleation of a new phase cluster in a pore proceeds in a mode when the energy barrier is absent, like spinodal decomposition of the unstable homogeneous system. Figure 7 presents the dependence of the heterogeneous spinodal position $x_{sh}$ on cone angle β and contact angle $\gamma_{0}$.

Is evident that the spinodal for heterogeneous nucleation is located nearer to the binodal as both the pore cone angle β (Figure 7a) and the macroscopic contact angle $\gamma_{0}$ (Figure 7b) yield its shift to decreasing values of *x*. If the equilibrium contact angle is equal to $\gamma_{0}=90{}^{{}^{\circ}}$, the heterogeneous spinodal coincides with the macroscopic one, i.e., $x_{sh}=x_{sp}^{\left(l\right)}$, like in the case of the homogeneous nucleation.

## 4. Heterogeneous Nucleation in a Conic Pore: Results

**Figure 8:**
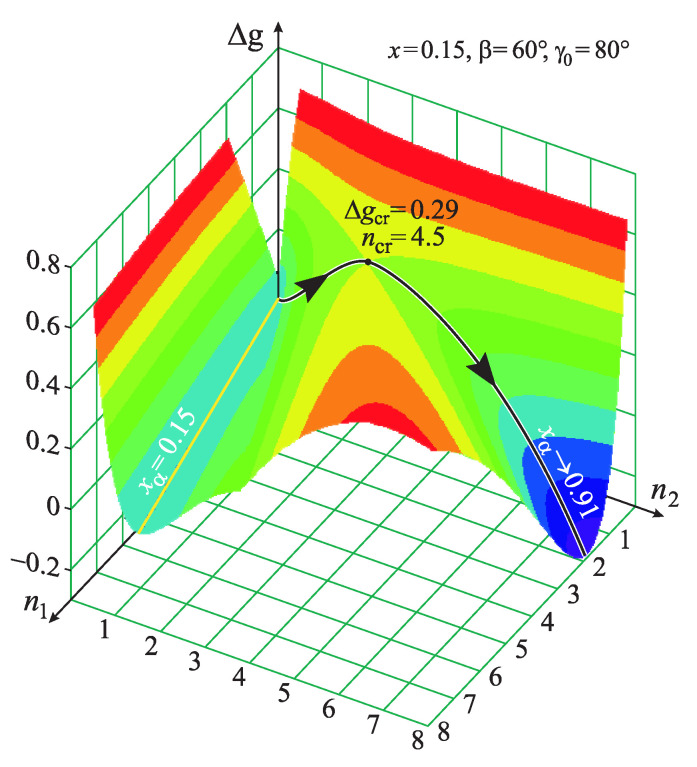
Shape of the Gibbs free energy of cluster formation in a metastable regular solution with x=0.15 ($x<x_{sh}\approx 0.178$) in a conic pore with the angle $\beta =60{}^{{}^{\circ}}$. The equilibrium contact angle is chosen equal to $\gamma_{0}=80{}^{{}^{\circ}}$.

For a metastable state of the initial solution, $x_{b}^{\left(l\right)}<x<x_{sp}^{\left(l\right)}$, the work of critical cluster formation in the space $\left(n_{1},n_{2}\right)$ has characteristic saddle points properties near to the parameters of the critical cluster, $\left(n_{1,cr},n_{2,cr}\right)$. The surface is shown in Figure 8 for the case of nucleation of a new phase cluster in a pore with an angle $\beta =60{}^{{}^{\circ}}$ and for an equilibrium contact angle $\gamma_{0}=60{}^{{}^{\circ}}$ and at the composition of the ambient phase equal to x=0.15. The “valley” at $x_{\alpha }=x=0.15$ corresponds to the initial state, and the saddle point to the critical cluster. Its parameters are determined by Equations (29). In the course of its growth, the new phase cluster passes through a saddle point. Finally, its composition tends to an equilibrium value nearly equal to the respective value on the right binodal $x_{\alpha }\to x_{b}^{\left(r\right)}\approx 0.91$.

**Figure 9:**
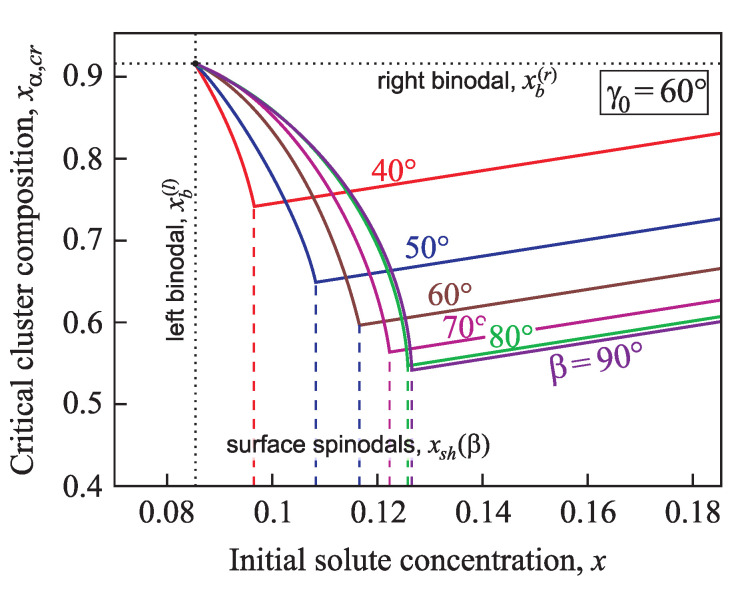
Composition of the critical cluster, $x_{\alpha ,cr}$, in dependence on supersaturation for nucleation in a conic pore with various angles $\beta =40{}^{{}^{\circ}},\;50{}^{{}^{\circ}},\;60{}^{{}^{\circ}},\;70{}^{{}^{\circ}},\;80{}^{{}^{\circ}}$, and $90{}^{{}^{\circ}}$. The equilibrium contact angle is chosen equal to $\gamma_{0}=60{}^{{}^{\circ}}$.

**Figure 10:**
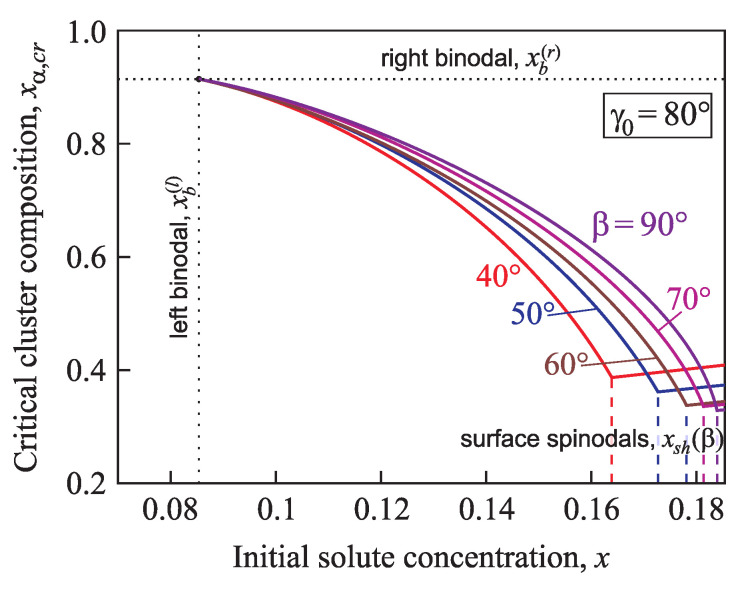
Composition of the critical cluster, $x_{\alpha ,cr}$, in dependence on supersaturation for nucleation in conic pores with various angles $\beta =40{}^{{}^{\circ}},\;50{}^{{}^{\circ}},\;60{}^{{}^{\circ}},\;70{}^{{}^{\circ}},\;80{}^{{}^{\circ}}$, and $90{}^{{}^{\circ}}$. Here the equilibrium contact angle is chosen equal to $\gamma_{0}=80{}^{{}^{\circ}}$.

The composition of a critical cluster, $x_{\alpha ,cr}$, is shown in Figure 9 and Figure 10 in dependence on the initial supersaturation for the case of nucleation in a conic pore with various angles $\beta \;=\;40^{{}^{\circ}},\;50^{{}^{\circ}},\;60^{{}^{\circ}},\;70^{{}^{\circ}},\;80^{{}^{\circ}},\;\mathrm{and}\;90^{{}^{\circ}}$ and two different values of the equilibrium contact angle $\gamma_{0}=60^{{}^{\circ}}$ (Figure 9) and $\gamma_{0}=80^{{}^{\circ}}$ (Figure 10). With an increase of the supersaturation from an initial value close to the binodal the concentration of the second component in the critical cluster, $x_{\alpha ,cr}$, decreases down to the minimum value $x_{\alpha ,0}$ at $x=x_{sh}\left(\gamma_{0}\right)$ (Figure 9 and Figure 10). If the supersaturation increases further, $x_{\alpha ,cr}$ grows linearly (see Equation (36) and Figure 6).

**Figure 11:**
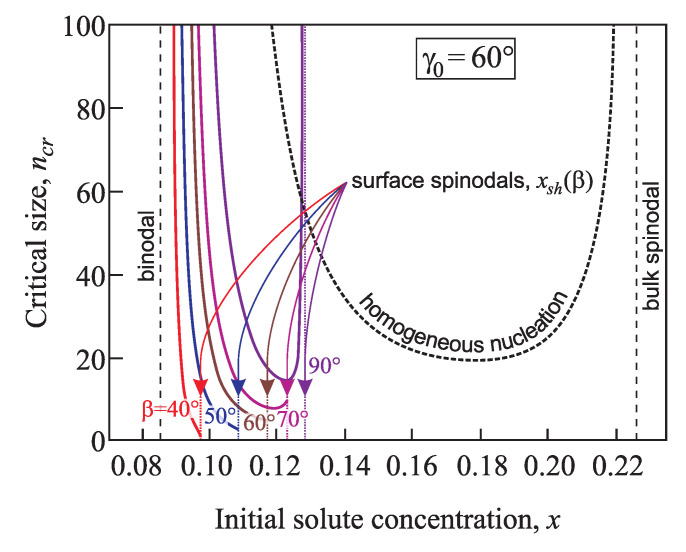
Critical cluster size, $n_{cr}$, as a function of the concentration for nucleation in conic pores with different angles $\beta =40{}^{{}^{\circ}},\;50{}^{{}^{\circ}},\;60{}^{{}^{\circ}},\;70{}^{{}^{\circ}}$, and $90{}^{{}^{\circ}}$. The equilibrium contact angle is taken as $\gamma_{0}=60{}^{{}^{\circ}}$. For comparison, the dashed line shows the dependence $n_{cr}\left(x\right)$ for homogeneous nucleation.

**Figure 12:**
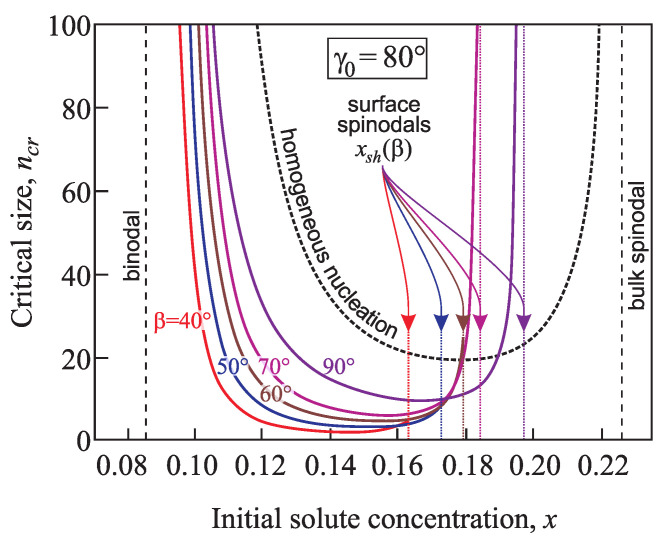
Critical cluster size, $n_{cr}$, as a function of the concentration for nucleation in conic pores with different angles $\beta =40{}^{{}^{\circ}},\;50{}^{{}^{\circ}},\;60{}^{{}^{\circ}},\;70{}^{{}^{\circ}}$, and $90{}^{{}^{\circ}}$. Here the equilibrium contact angle is taken equal to $\gamma_{0}=80{}^{{}^{\circ}}$. For comparison, the dashed line shows the dependence $n_{cr}\left(x\right)$ for homogeneous nucleation.

According to the classical nucleation theory the size of a critical cluster tends to infinity for initial phase composition approaching the binodal. With an increase of the supersaturation grows the critical cluster size decreases. At $x>x_{sh}$, the critical size in its classical interpretation does not exist anymore because cluster growth can proceed without overcoming a thermodynamic potential barrier starting from n=0 (or, in a more precise formulation, starting with one structural unit). However, in terms of the generalized Gibbs approach, in contrast to the classical theory and in agreement with density functional computations [7,8], this decrease in size may be followed by an increase with a further increase of the supersaturation (Figure 11 and Figure 12). Consequently, the transition from metastable to thermodynamically unstable states proceeds here in a quite different way. As one consequence it follows that near to the spinodal the formation of critical clusters will, in general, not proceed via the saddle point of the thermodynamic potential surface but via a ridge point (for details see [17,18]).

For the equilibrium contact angle $\gamma_{0}=60{}^{{}^{\circ}}$ and small values of the cone pore angles $\beta \;=\;40{}^{{}^{\circ}},\;50{}^{{}^{\circ}},\;\mathrm{and}\;60{}^{{}^{\circ}}$, along with the increase of the supersaturation the critical cluster size decreases monotonically from infinity at the binodal up to values of *x* at the spinodal for heterogenous nucleation $x=x_{sh}$ (Figure 11). Then it exhibits a discontinuity and becomes equal to zero at further increase of the supersaturation. For planar solids surfaces $\beta =90{}^{{}^{\circ}}$, the decrease of the critical cluster size with increasing supersaturation is followed by its further increase. This increase is then also followed by a similar discontinuity at $x=x_{sh}$.

When the equilibrium contact angle equals $\gamma_{0}=80{}^{{}^{\circ}}$ and the cone pore angle has values in the range $\beta =40\;–90^{{}^{\circ}}$ the dependence of the critical cluster size on supersaturation is non-monotonic: first $n_{cr}$ decreases from infinity at the binodal, then the decrease is followed by its growth up to $x=x_{sh}$, and for $x\geq x_{sh}$ the critical cluster size becomes equal to zero (Figure 12).

**Figure 13:**
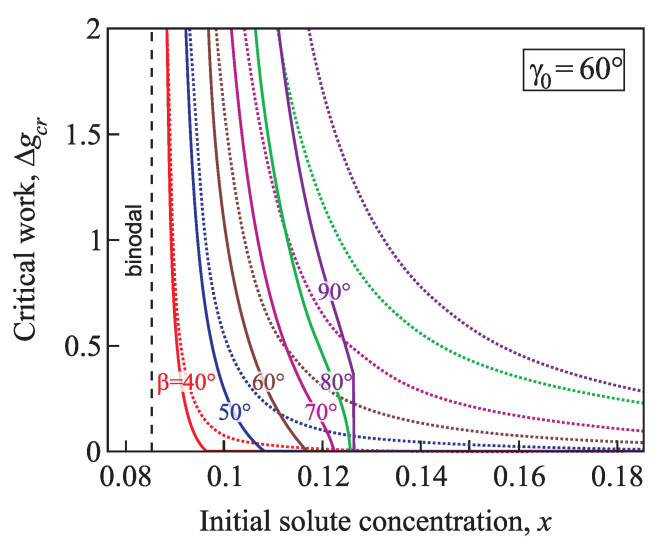
Normalized work of formation of a critical cluster, $\Delta g_{cr}$, as function of the concentration for nucleation in conic pores with various angles $\beta =40{}^{{}^{\circ}},\;50{}^{{}^{\circ}},\;60{}^{{}^{\circ}},\;70{}^{{}^{\circ}},\;80{}^{{}^{\circ}}$, and $90{}^{{}^{\circ}}$; the equilibrium contact angle is $\gamma_{0}=60{}^{{}^{\circ}}$. For a comparison, the dotted lines show the function $\Delta g_{cr}\left(x\right)$ calculated via the classical nucleation theory for conic pores.

**Figure 14:**
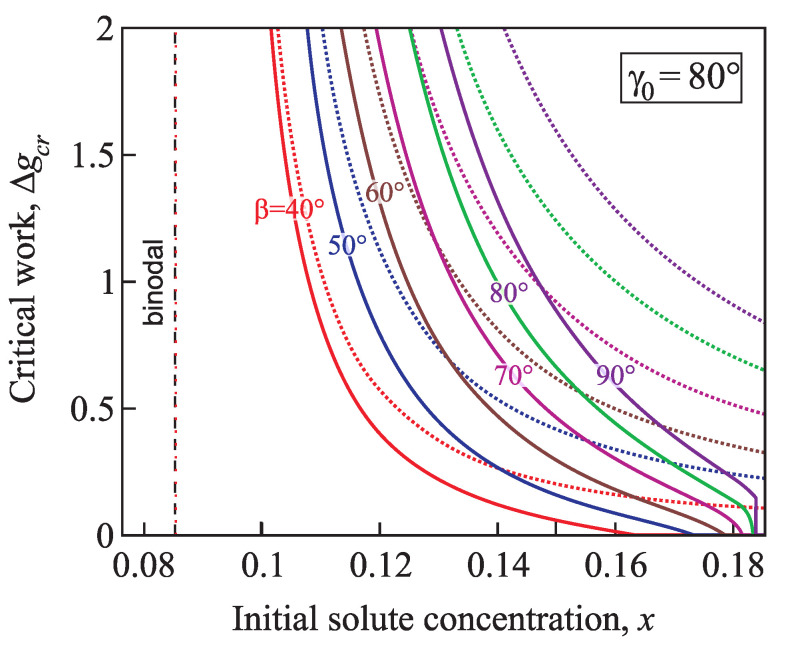
Normalized work of formation of a critical cluster, $\Delta g_{cr}$, as function of the concentration for nucleation in conic pores with various angles $\beta =40{}^{{}^{\circ}},\;50{}^{{}^{\circ}},\;60{}^{{}^{\circ}},\;70{}^{{}^{\circ}},\;80{}^{{}^{\circ}}$, and $90{}^{{}^{\circ}}$; the equilibrium contact angle is $\gamma_{0}=80{}^{{}^{\circ}}$. For comparison, the dotted lines show the function $\Delta g_{cr}\left(x\right)$ calculated via the classical nucleation theory for conic pores.

Figure 13 and Figure 14 illustrate the normalized work of formation of a critical cluster, $\Delta g_{cr}=\left(\Delta G_{cr}/G_{\sigma }\right)$, in dependence on supersaturation for nucleation in conic pores with various angles $\beta =40^{{}^{\circ}},\;50^{{}^{\circ}},\;60^{{}^{\circ}},\;70^{{}^{\circ}},\;80^{{}^{\circ}}$, and $90^{{}^{\circ}}$, the equilibrium contact angle is $\gamma_{0}=60^{{}^{\circ}}$ (Figure 13) and $\gamma_{0}=80^{{}^{\circ}}$ (Figure 14). The work of formation of a cluster decreases from infinity at the binodal, and for $x\geq x_{sh}$ it becomes equal to zero. The less the cone pore angle β and the contact angle $\gamma_{0}$ are, the faster the work of a critical cluster formation decreases.

The work of critical cluster formation determines widely the steady-state nucleation rate, *J*. It can be expressed generally as (see, for example [1,4])

(38) $$J=J_{0}\exp\left(-\frac{\Delta g_{cr}G_{\sigma }}{k_{B}T}\right).$$

The pre-exponential factor, $J_{0}$, is determined by the diffusion coefficients of the solution and by the number of possible nucleation centers per unit area. The quantity $G_{\sigma }$ is determined by Equation (25).

**Figure 15:**
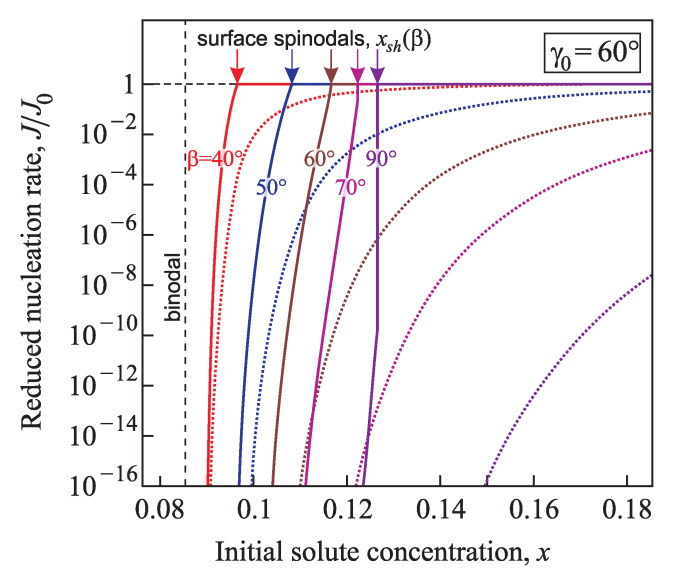
Comparison of the normalized steady-state nucleation rates, $J/J_{0}$, computed via the generalized Gibbs approach (solid lines) and using classical nucleation theory (dotted line) in conic pores with various angles $\beta =40{}^{{}^{\circ}},\;50{}^{{}^{\circ}},\;60{}^{{}^{\circ}},\;70{}^{{}^{\circ}}$, and $90{}^{{}^{\circ}}$. The equilibrium contact angle is taken as $\gamma_{0}=60{}^{{}^{\circ}}$.

**Figure 16:**
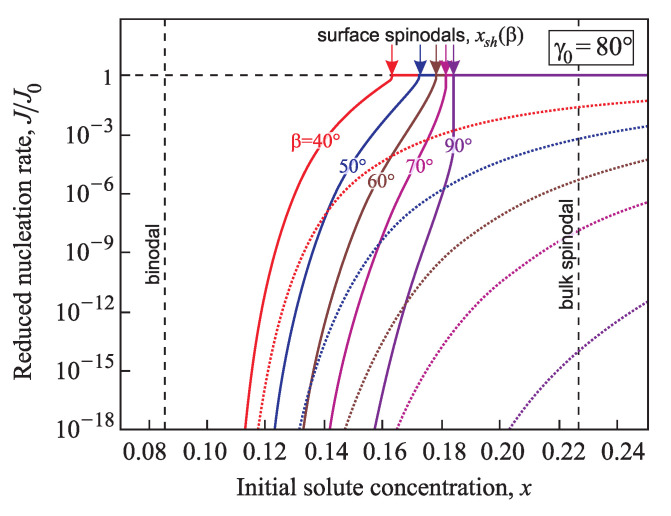
Comparison of the normalized nucleation rates, $J/J_{0}$, determined via the generalized Gibbs approach (solid lines) and in terms of the classical nucleation theory (dotted lines) in conic pores with various angles $\beta =40{}^{{}^{\circ}},\;50{}^{{}^{\circ}},\;60{}^{{}^{\circ}},\;70{}^{{}^{\circ}}$, and $90{}^{{}^{\circ}}$. The equilibrium contact angle is taken here $\gamma_{0}=80{}^{{}^{\circ}}$.

Figure 15 and Figure 16 supply us with a comparison of the normalized nucleation rates, $J/J_{0}$, in conic pores with various angles $\beta =40{}^{{}^{\circ}},\;50{}^{{}^{\circ}},\;60{}^{{}^{\circ}},\;70{}^{{}^{\circ}}$, and $90{}^{{}^{\circ}}$ determined within the generalized (solid lines) and via the classical Gibbs (dotted line) approaches in the case of good wettability ($\gamma_{0}=60{}^{{}^{\circ}}$ and $80^{{}^{\circ}}$). The calculations were performed for a temperature $T=0.7T_{c}$ with $T_{c}=1143$ K and the parameters $G_{\sigma }=61.6\;k_{B}T$ and $R_{\sigma }=3.087a$ with $a=3.65\times 10^{-10}$ m. The nucleation rate calculated via the generalized Gibbs approach is much higher than the results obtained via the classical theory. With the increase of the supersaturation it reaches the maximum value, $J_{0}$, at $x\geq x_{sh}\left(\gamma_{0},\beta \right)$.

## 5. Conclusions

The generalized Gibbs approach applied to the description of the precipitation in a binary regular solution on a rough solid surface (conic pore) results as a whole in similar conclusions as obtained by us earlier in the analysis of heterogeneous nucleation in a one-component van der Waals liquid [13]: the presence of heterogeneous nucleation centers can effectively result in a shift of the spinodal from the value $x=x_{sp}^{\left(l\right)}$, as shown in Figure 7, to smaller values of the concentration, $x=x_{sh}\leq x_{sp}^{\left(l\right)}$. Therefore, the concentration range $x_{b}^{\left(l\right)}<x<x_{sh}$ of the initial solution we can consider as metastable with respect to heterogeneous nucleation, and the concentration range $x>x_{sh}$ as thermodynamically unstable. This result has the consequence that the range of metastable states decreases at the expense of an increase of the instability region, resulting in intensification of the nucleation rate. This effect became stronger with a decrease of the cone angle of the pore and the equilibrium contact angle. In line with the general result obtained in [19] for the case of homogeneous nucleation, also in heterogeneous nucleation the generalized Gibbs approach yields lower values of the work of critical cluster formation and higher values of the steady-state nucleation rates as compared to the results obtained via Gibbs classical treatment.

Actually, the surface roughness is not uniform. In frame of the studied approach the roughness state of a surface may differ in depth of the cones and their cone angles. Its account may be approximated as a spread in these parameters within some model distribution. As can be seen from the comparison of plots in Figure 7 the differences in cone angle do influence the cluster critical size and with it the nucleation probability. The lesser the cone angle is the more important is the role of heterogeneous nucleation. In addition, the dependence of the nucleation rate on cone parameters is exponential. It means that the nucleation preferentially proceeds in pores with the lowest cone angle. The cone depth may also influence the nucleation when it is too low to form a viable cluster in it. This situation can happen on rather smooth surfaces. As it follows from Figure 7 more influential is the change of the equilibrium contact angle but it is rather the case of different surface materials, say composites or metamaterials.
